# Diagnostic Test Accuracy of Apparent Diffusion Coefficient in Evaluation of Breast Cancer Lymph Node Metastasis: A Systematic Review and Meta‐Analysis

**DOI:** 10.1002/cnr2.70395

**Published:** 2025-11-08

**Authors:** Amirmohammad Azizzadeh, Fahimeh Zeinalkhani, Peyman Kamali Hakim, Aida Mousavi

**Affiliations:** ^1^ Student's Scientific Research Center Tehran University of Medical Sciences Tehran Iran; ^2^ Student Research Committee Tabriz University of Medical Sciences Tabriz Iran; ^3^ Advanced Diagnostic and Interventional Radiology Research Center (ADIR) Tehran University of Medical Science Tehran Iran; ^4^ Department of Radiology Iran University of Medical Sciences Tehran Iran

**Keywords:** breast neoplasms, diffusion magnetic resonance imaging, lymphatic metastasis, meta‐analysis, sensitivity and specificity

## Abstract

**Aims:**

This study aimed to evaluate the diagnostic accuracy of the apparent diffusion coefficient (ADC) derived from diffusion weighted imaging (DWI) for detecting lymph node metastasis in breast cancer.

**Methods:**

A systematic review and meta‐analysis was conducted following the PRISMA‐DTA guidelines. PubMed, Web of Science, PROQUEST, and EMBASE were searched. ADC values for suspected lymph nodes in breast cancer patients were assessed, with histopathology as the reference test. Data were analyzed using a bivariate meta‐analytical model with Meta‐DiSc 2.0 and STATA 17. Meta‐regression and subgroup analysis were conducted to assess heterogeneity.

**Results:**

Twenty‐six studies encompassing 2828 participants were included. The pooled sensitivity and specificity of ADC value for detecting lymph node metastasis were 88.6% (CI = 82.4%–92.8%) and 83.6% (CI = 78.0%–88.1%), respectively. The area under the HSROC curve was 0.92 (CI = 0.89–0.94), indicating excellent diagnostic ability. Moderate heterogeneity was detected (prediction area = 0.369; bivariate *I* ^2^ = 69.9%). Field of view and retrospective study designs were identified as factors linked with higher specificity. Selection of largest nodes for analysis and participants' age were factors associated with higher sensitivity. The pooled ADC value was 1.272 × 10^−3^ mm^2^/s (CI = 1.150–1.394) for benign nodes and 0.874 × 10^−3^ mm^2^/s (CI = 0.773–0.974) for metastatic nodes.

**Conclusion:**

ADC value is a highly accurate non‐invasive diagnostic marker for lymph node metastasis in breast cancer. We recommend acquisition of DWI in women less than 51 years old, with the highest *b*‐value of 1000 s/mm^2^, repetition time ≥ 8500 ms, field of view ≥ 350 mm, and selection of the largest node for analysis.

## 1 Introduction

Breast cancer, the most frequently diagnosed cancer among women, is expected to increase by 134% worldwide and the most significant increase is anticipated to be in low‐income and lower‐middle‐income nations [1]. A key predictor of recurrence, prognosis, and survival among breast cancer patients is lymph node (LN) metastasis. The 5‐year rate of survival for localized disease is 14% higher than that for patients with regional nodal metastases [2]. The count and locality of metastatic nodes also determine breast cancer stage [3]. Surgical complexity and the requirement for further systemic or radiation therapy are often dictated by lymphatic node involvement. Therefore, the assessment of axillary LNs is a critical element in staging and treatment selection [3].

The current established gold standard for evaluating LN metastasis of breast cancer consists of sentinel lymph node biopsy (SLNB) or axillary lymph node dissection (ALND). Although SLNB has largely replaced routine dissection of axillary LNs in most patients and is relatively less invasive, it is still associated with complications such as lymphedema, pain, and reduced limb function [4, 5]. As a result, efforts are underway to develop less disruptive diagnostic approaches.

In response to the growing clinical demand for noninvasive techniques that can preoperatively diagnose LN metastasis, imaging modalities are currently undergoing rapid development. Magnetic resonance imaging (MRI) has found its footing in the evaluation of breast cancer in the areas of systemic treatment response monitoring, multifocal cancers, and the definition of disease extent. The guidelines of the National Comprehensive Cancer Network (NCCN) call for MRI with contrast for these evaluations [6, 7, 8]. MRI with contrast enhancement has been shown to be highly sensitive for LN metastasis staging but lacks specificity [9]. Therefore, falsely positive results on breast MRI scans are common [6]. The use of intravenous contrast agents is another limiting factor for this modality.

Diffusion weighted imaging (DWI) has demonstrated encouraging results in the detection of breast cancer [10]. DWI is an MRI technique that makes use of the natural atomic‐level movement of water molecules, known as Brownian motion, to create image contrast. In biological tissues, the movement of water is not completely free and is influenced by cell density, membrane integrity, and the presence of barriers such as fibers or macromolecules. By applying special gradient pulses during MRI, DWI can detect these tiny differences in water movement and translate them into differences in image brightness. From these images, a parameter called the apparent diffusion coefficient (ADC) is calculated. The ADC value provides a numerical measure of how easily water molecules can move within a particular tissue. It is called “apparent” because the measured movement reflects both true molecular diffusion and the restrictions caused by the microscopic tissue environment. This calculated parameter allows for precise quantification of a given region of interest (ROI). ADC values are usually expressed in standardized units (×10^−3^ mm^2^/s). Higher ADC values indicate that water molecules can move more freely, as in fluids or tissues with low cellular density. Lower ADC values indicate more restricted movement, which typically occurs in tissues that are tightly packed with cells or structural barriers. In this way, DWI and ADC together provide a non‐invasive means of assessing the microscopic structure of tissues by simply observing how water molecules move within them [11].

The standard protocol and parameters for acquiring DWI of axillary LNs has not yet been established, leading to variations that have resulted in a broad spectrum of ADC values and varying reported diagnostic indices. This systematic review and meta‐analysis aimed to synthesize the existing evidence on the diagnostic accuracy of ADC value in detecting breast cancer LN metastasis, thereby providing a comprehensive evaluation of its clinical applicability.

## 2 Methods

### 2.1 Study Objective

This systematic review and meta‐analysis was conducted in accordance with the Cochrane Handbook of Diagnostic Test Accuracy Systematic Reviews, and the PRISMA‐DTA reporting guidelines. The research protocol was registered in PROSPERO (CRD42024556477). The Internal Review Board granted ethical and methodological approval (approval ID: IR.TUMS.IKHC.REC.1403.244).

### 2.2 Search Strategy

The research question was formulated using the PIRD framework (population, index test, reference test, and diagnosis), which is specifically tailored for studies on diagnostic test accuracy. The research question can be stated as follows: “What is the diagnostic test accuracy of the ADC value derived from DWI for detecting axillary LN metastasis in breast cancer?”

A detailed search was carried out in the electronic databases of PubMed, Web of Science, ProQuest, preprint indexes (via WOS), and EMBASE. A manual search was performed using Google Scholar. Additional studies were identified by checking the reference lists of previous reviews. No language restrictions were applied, and articles published up to October 2023 were evaluated. The search strategy was developed using a combination of structured and free keywords. The search syntax included “breast cancer,” “lymph node metastasis,” and “ADC values.” The complete search syntax can be seen in Tables S1–S3.

### 2.3 Eligibility Screening

The following criteria were used to include studies: (1) included patients diagnosed with breast cancer; (2) assessed the ADC obtained from DWI for suspected LNs; (3) histopathology served as the reference test; and (4) reported diagnostic test accuracy indices.

The exclusion criteria were as follows: (1) provided information did not allow for the construction of a 2 × 2 table; (2) nonstandard MRI protocols; (3) animal studies; and (4) reviews, case reports, and correspondences.

The search results were consolidated using Zotero software. The documents were transferred to Rayyan software for deduplication and screening. During the initial screening, two blinded reviewers (A.A. and A.M.) independently assessed the titles and abstracts to determine their suitability according to the predefined criteria. During the second screening stage, two reviewers (A.A. and A.M.) independently reviewed the full text of the articles. A third reviewer (F.Z.) evaluated the decisions, and any disagreements were resolved through discussion.

### 2.4 Data Extraction

A data extraction form was designed and used. Two researchers (A.A. and A.M.) independently extracted the relevant data from the included studies. In cases with incomplete data, the authors were contacted. If the data were displayed in a graphical format, reliable estimations were obtained using WebPlotDigitizer. Estmeansd program was used to convert median and interquartile range into mean and standard deviation (SD). True and false positive and negative counts were calculated using reported sensitivity and specificity. The following data were collected.

- Publication details: Author, DOI, and publication year
- Methodology: location of the study, study period, study design
- Participant characteristics: patient count, axilla count, node count, and patient age
- Imaging specifications: MRI field strength, slice thickness, repetition time (TR), field of view (FOV), MRI position, breast coil, *b*‐values, use of contrast before DWI
- Reference test, LN selection method, and analysis type
- Primary outcome measures: true positive, true negative, false positive, and false negative counts
- Secondary outcomes: Mean and SD of ADC values of metastatic and benign nodes, ROC area under the curve (AUC) and standard error (SE)

### 2.5 Quality and Risk of Bias Assessment

The included studies' applicability and bias risk were evaluated using the Quality Assessment of Diagnostic Accuracy Studies 2 (QUADAS‐2) tool. Two reviewers evaluated each study separately and any disagreements were addressed by a third reviewer. Robvis web‐app was used to trace the risk of bias plots.

### 2.6 Data Synthesis and Analysis

Summary diagnostic test accuracy statistics along with 95% confidence intervals (CI) were calculated using the bivariate random effects meta‐analysis model with Meta‐DiSc 2.0. A summary receiver operating characteristic (SROC) curve was generated with STATA 17 using the MIDAS package. The AUC served as an index of classification accuracy. Both software solutions utilize the models developed by Reitsma and Chu [12, 13].

Heterogeneity was assessed by estimates of the *Q* test, bivariate *I* ^2^, and the 95% prediction ellipse area. The threshold effect was assessed based on the proportion of variation attributable to the threshold using the Spearman rank correlation. Meta‐regression and subgroup analysis were used to investigate factors contributing to heterogeneity. A significant criterion of a *p*‐value < 0.05 was chosen. Publication bias was evaluated using Deek's funnel plot regression test. A *p*‐value of less than 0.1 indicates significant asymmetry.

Meta‐analysis of study‐level AUC was conducted in MedCalc 22.03 using the random effects model. Pooled AUC value with CI and *I* ^2^ estimate was reported. Additionally, ADC values of non‐metastatic and metastatic LNs were extracted and pooled separately. ADC values between these two groups were compared using a mean difference meta‐analysis. Pooled mean and CI, mean difference and CI, and heterogeneity parameters (*I* ^2^, *Q* test, and prediction interval) were calculated. Violin plots for the distribution of ADC values were generated. Analysis was conducted and the results were plotted using OnlineMeta software.

The summary of findings table was prepared using GRADEpro. Summary parameters were reported for three prevalence points, each representing different stages of breast cancer.

## 3 Results

### 3.1 Study Selection

**FIGURE 1:**
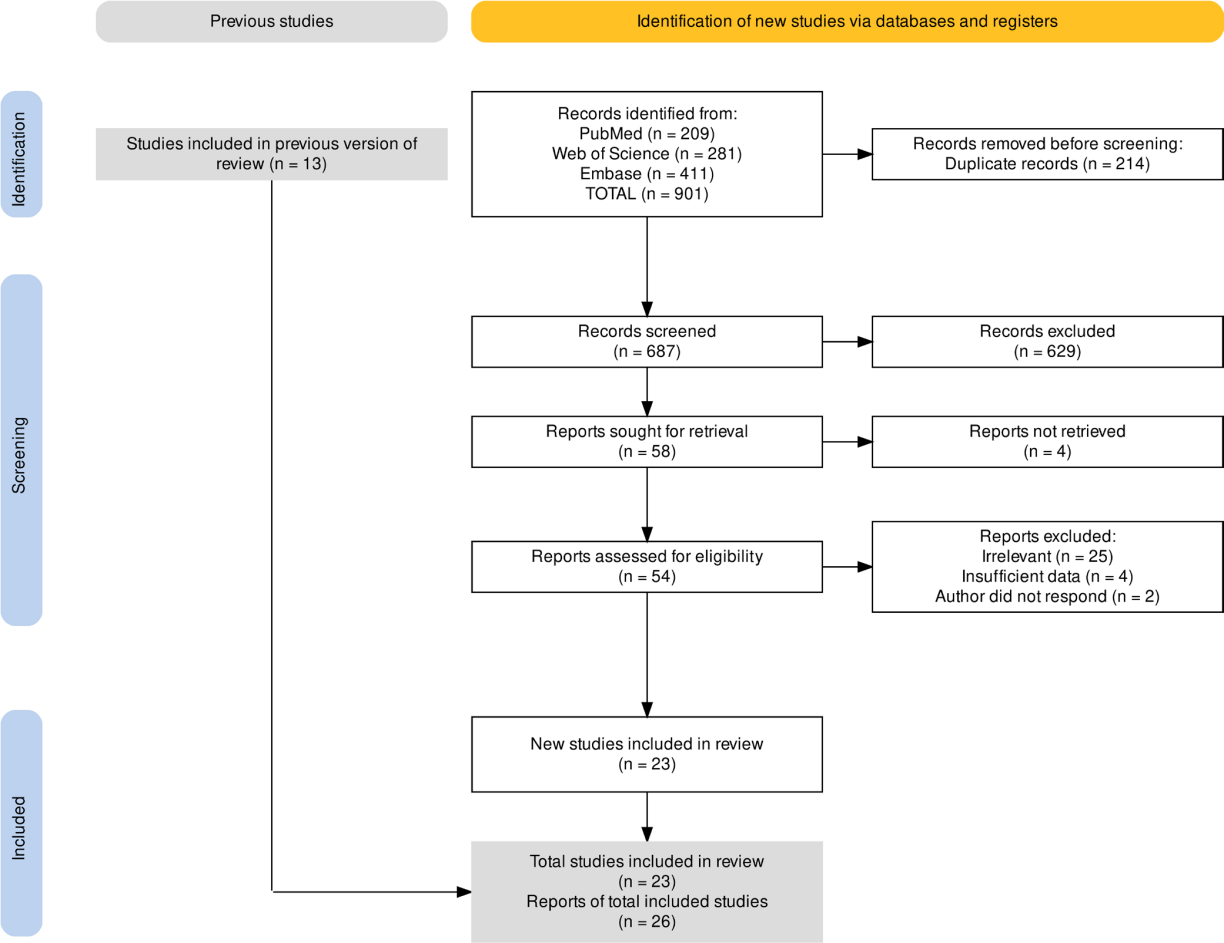
PRISMA flow diagram.

A total of 687 unique records were identified through our search, and 13 more studies were added through manual searching. Ultimately, 26 records were eligible for inclusion [14, 15, 16, 17, 18, 19, 20, 21, 22, 23, 24, 25, 26, 27, 28, 29, 30, 31, 32, 33, 34, 35, 36]. Two studies [23, 27] included different imaging modes, and another study [32] reported patient‐wise and node‐wise results. Figure 1 presents the detailed flowchart of the screening process.

### 3.2 Study Characteristics

In total, 26 records with a median sample size of 63.5, encompassing a sample size of 2828 (1332 cases and 1496 non‐cases) were included. The prevalence of metastasis was found to be 47.1%. The studies were conducted between 2012 and 2022. Sixteen studies utilized a prospective study design, and 10 studies had a retrospective design. Nineteen studies utilized MRI scanners with a magnet strength of 1.5 T, four studies used 3 T machines, and three studies employed a combination of 1.5 and 3 T machines. The *b*‐values ranged from zero to 1000 s/mm^2^, and seven studies utilized more than two *b*‐values. Eleven studies utilized SLNB or ALND for the reference test, while the remaining 16 studies employed a combination of histopathological evaluations. Tables 1, S4 and S5 include detailed study characteristics.

**TABLE 1:** Study characteristics.

ID	Author	Year	Location	Study design	Number of patients	Reference test	Mean age	Benign ADC (SD)	Metastatic ADC (SD)	AUC (SE)
1	Başara [14]	2013	Türkiye	Prospective	110	Histopathology	46.44	1.39 (0.30)	1.00 (0.30)	0.814 (0.041)
2	Chung [15]	2014	South Korea	Retrospective	110	SLNB or ALND	50.75	1.40 (0.19)	0.69 (0.10)	0.968 (0.016)
3	De Cataldo [16]	2020	Italy	Prospective	107	SLNB or ALND	51	1.30 (0.55)	0.62 (0.15)	0.876 (0.035)
4	Duran [17]	2021	Türkiye	Retrospective	102	Histopathology	52	1.17 (0.20)	0.77 (0.19)	0.929 (0.042)
5	Elmesidy [18]	2021	Egypt	Prospective	77	Histopathology	50	1.12 (0.32)	0.84 (0.28)	0.754 (0.055)
6	Fardanesh [19]	2022	United States	Retrospective	217	SLNB or ALND	52	1.28 (0.45)	0.94 (0.24)	NA
7	Fornasa [20]	2012	Italy	Prospective	43	SLNB or ALND	58.49	1.49 (0.38)	0.88 (0.21)	0.923 (0.045)
8	Guvenc [21]	2019	United States	Retrospective	85	Histopathology	53	1.41 (0.21)	0.89 (0.18)	0.960 (0.021)
9	Hasanzadeh [22]	2017	Iran	Prospective	30	SLNB or ALND	51.06	1.10 (0.23)	0.82 (0.10)	NA
10	He (a) [23]	2012	China	Prospective	136	Histopathology	44	1.76 (0.35)	1.37 (0.18)	0.840 (0.025)
11	He (b) [23]	2012	China	Prospective	136	Histopathology	44	1.55 (0.31)	1.18 (0.12)	0.870 (0.022)
12	Ismail [24]	2014	Egypt	Prospective	44	Histopathology	55	1.42 (0.57)	0.79 (0.23)	NA
13	Kamitani [25]	2013	Japan	Retrospective	108	SLNB or ALND	54.9	0.92 (0.22)	1.08 (0.18)	NA
14	Kim [26]	2014	South Korea	Retrospective	252	Histopathology	51.6	1.27 (0.32)	0.91 (0.30)	0.815 (0.030)
15	Kurt (a) [27]	2022	Türkiye	Retrospective	66	SLNB or ALND	49.75	1.52 (0.32)	1.03 (0.38)	0.960 (0.023)
16	Kurt (b) [27]	2022	Türkiye	Retrospective	66	SLNB or ALND	49.75	1.53 (0.32)	1.05 (0.48)	0.940 (0.028)
17	Latif [28]	2016	Egypt	Prospective	30	Histopathology	46.5	1.40 (0.30)	0.70 (0.16)	0.944 (0.039)
18	Luo [29]	2013	China	Prospective	36	Histopathology	53	1.04 (0.26)	0.79 (0.14)	0.833 (0.045)
19	Razek [30]	2016	Egypt	Prospective	34	Histopathology	51	1.58 (0.14)	1.08 (0.21)	0.974 (0.018)
20	Scaranelo [31]	2012	Canada	Prospective	61	SLNB or ALND	53	0.72 (0.17)	0.67 (0.50)	0.743 (0.063)
21	Schipper (a) [32]	2015	Netherlands	Prospective	50	SLNB or ALND	60	0.75 (0.26)	0.67 (0.34)	0.580 (0.069)
22	Schipper (b) [32]	2015	Netherlands	Prospective	50	SLNB or ALND	60	0.75 (0.26)	0.67 (0.34)	0.740 (0.090)
23	Yamaguchi [33]	2015	United States	Retrospective	36	Histopathology	51.72	1.03 (0.25)	0.75 (0.15)	0.884 (0.061)
24	Yilmaz [34]	2019	Türkiye	Retrospective	43	Histopathology	44.42	1.15 (0.45)	0.76 (0.20)	NA
25	Zahran [35]	2022	Egypt	Prospective	29	Histopathology	47.43	0.99 (0.26)	0.68 (0.15)	0.878 (0.051)
26	Zaiton [36]	2016	Egypt	Prospective	40	Histopathology	32	1.53 (0.60)	0.86 (0.90)	0.924 (0.018)

### 3.3 Quality Assessment

**FIGURE 2:**
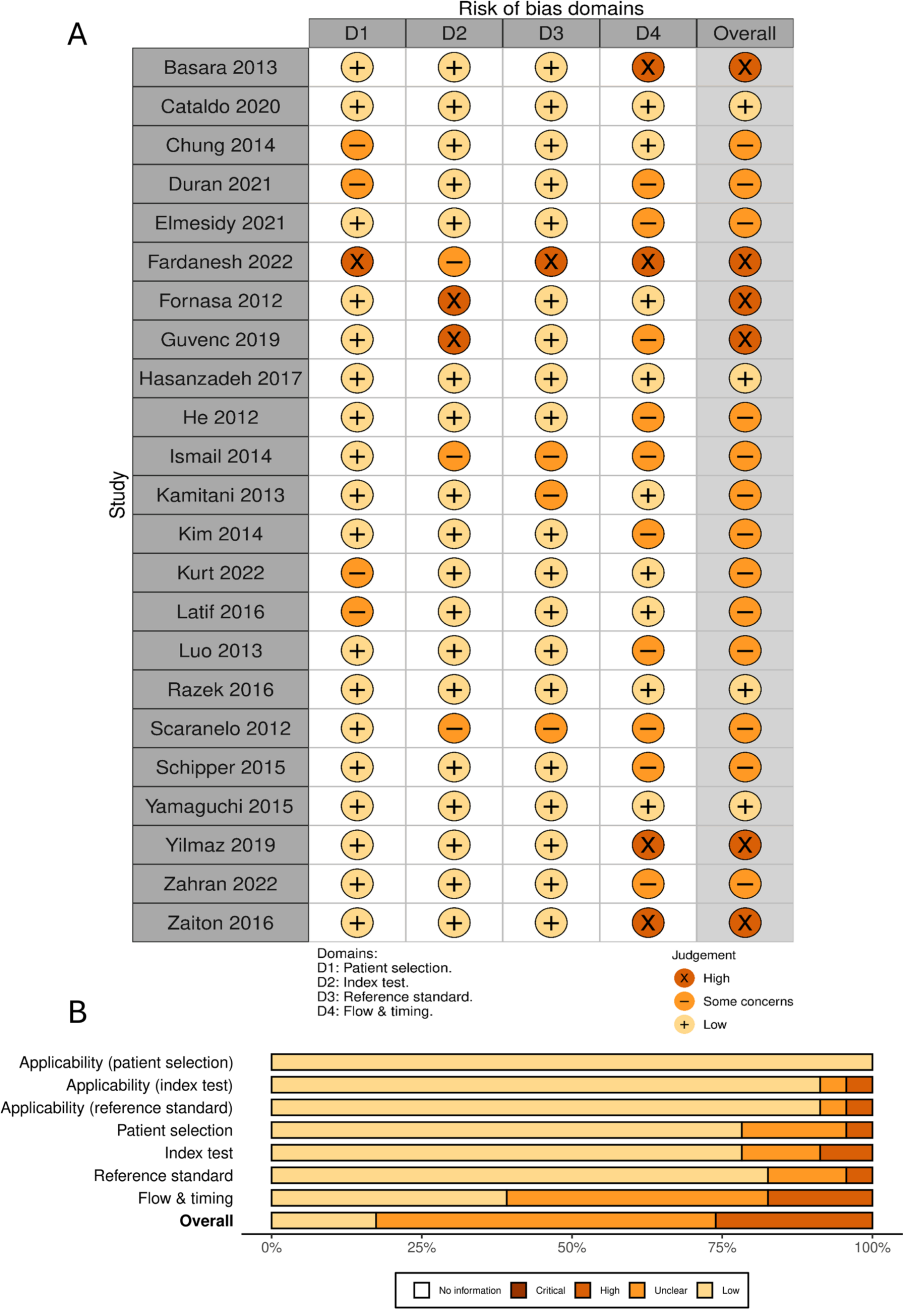
Risk of bias assessment using QUADAS‐2. (A) Authors' judgment for the included studies in each domain of QUADAS‐2; (B) Overall and domain‐specific summary of risk of bias using QUADAS‐2.

A visual representation of the risk of bias is shown in Figure 2. Applicability concerns were predominantly low. In general, the included studies' methodological quality was considered unclear. Most studies scored well on the patient selection, index test, and reference standard domains. However, in the flow and timing domain, four studies had a high risk of bias and 11 more were marked as unclear. This ambiguity arose from concerns regarding the variability of the reference standard in studies that employed a combination of histopathological techniques as diagnostic references. Additionally, some studies were unclear regarding the gap between the index test and the reference standard.

### 3.4 Diagnostic Test Accuracy

**FIGURE 3:**
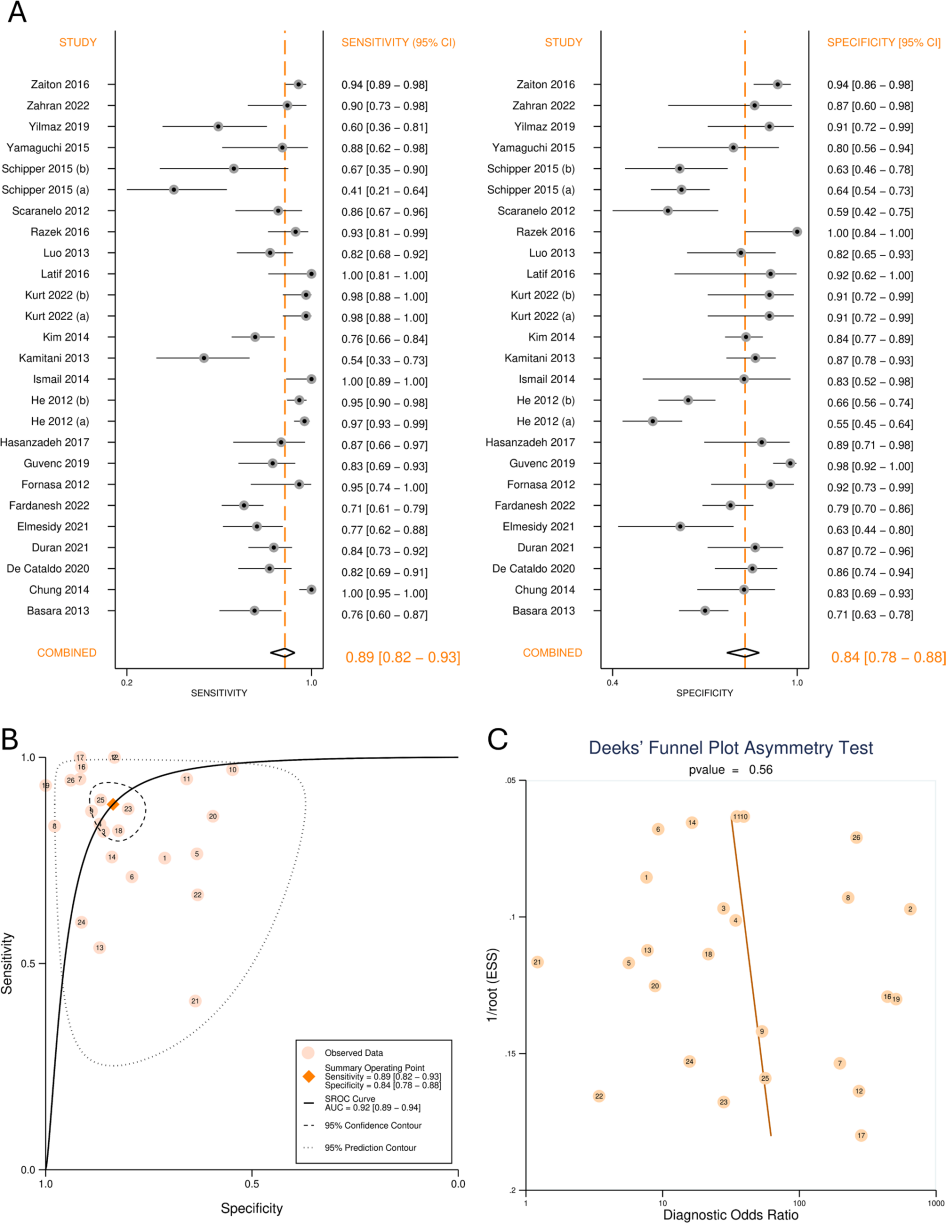
Diagnostic test accuracy of apparent diffusion coefficient for detection of breast cancer lymph node metastasis. (A) Forest plot of sensitivity and specificity; (B) Summary receiver operating characteristics curve (SROC); (C) Deek's funnel plot for publication bias.

The forest plot in Figure 3 depicts sensitivity, specificity, and CI. The model reported a pooled sensitivity of 88.6% (CI = 82.4%–92.8%), and specificity of 83.6% (CI = 78.0%–88.1%). Summary estimates of meta‐analysis and heterogeneity are provided in Table 2. The SROC plot is presented in Figure 3. Diagnostic ability was deemed excellent by the SROC AUC of 0.92 (CI = 0.89–0.94).

**TABLE 2:** Summary of meta‐analysis and heterogeneity estimates.

	Estimate	95% Lower CI	95% Upper CI
Sensitivity	88.6%	82.4%	92.8%
Specificity	83.6%	78.0%	88.1%
Diagnostic odds ratio	39.64	20.22	77.72
Positive likelihood ratio	5.413	3.922	7.470
Negative likelihood ratio	0.137	0.086	0.216
False positive rate	0.164	0.119	0.220
SROC AUC	0.92	0.89	0.94
Pretest probability (prevalence)	0.470		
*Q* value	98.03		
*p* value for *Q* test	0.000		
Bivariate *I* ^2^	69.9%		
Area 95% prediction ellipse	0.369		
Correlation	0.233		
Threshold effect proportion	0.060		

Abbreviation: CI, Confidence Interval.

### 3.5 Publication Bias

The deek's funnel plot is shown in Figure 3. A symmetrical shape and non‐significant regression slope (*p*‐value = 0.56) were suggestive of the absence of publication bias.

### 3.6 Heterogeneity and Meta‐Regression

Heterogeneity was evaluated in several steps. *Q*‐value of 98.03 (*p*‐value < 0.001) indicated the presence of variation among the observed effect sizes which was also apparent in the forest plot. The bivariate *I* ^2^ was estimated to be 69.9%, which represents the proportion of variance in observed effects due to variations in true effects rather than chance. The 95% prediction ellipse area represents the amount of variation in true effect size. Although no official cut‐off has been suggested, generally values below 0.2 may be considered low and values above 0.8 may be considered high [37, 38]. Overall, moderate heterogeneity, mainly due to variations in true effect size, can be concluded.

To identify the source of heterogeneity, the threshold effect was first considered. The proportion of variation due to threshold was 0.06 and thus the threshold effect was not identified as a source of heterogeneity. To evaluate the source of heterogeneity, meta‐regression and subgroup analysis were performed. The results are shown in Tables 3 and S6.

**TABLE 3:** Subgroup analysis and meta‐regression results of the bivariate model.

Covariates	Categories	Sensitivity (CI)	*p*	Specificity (CI)	*p*
Number of *b*‐values	More than two	83.6% (66.3% to 92.9%)	0.326	77.1% (63.2% to 86.8%)	0.186
	Two	89.9% (83.4% to 94.0%)		85.3% (79.5% to 89.6%)	
Highest *b*‐value	1000	88.3% (77.2% to 94.4%)	0.933	88.4% (81.1% to 93.1%)	0.080
	Less than 1000	88.8% (80.6% to 93.7%)		79.9% (72.2% to 85.9%)	
Study design	Prospective	89.6% (82.1% to 94.2%)	0.594	79.1% (71.4% to 85.2%)	0.049
	Retrospective	86.7% (74.9% to 93.5%)		88.6% (82.1% to 92.9%)	
DWI before contrast	No	92.6% (67.8% to 98.7%)	0.584	89.8% (70.8% to 97.0%)	0.391
	Yes	88.1% (81.6% to 92.6%)		82.9% (77.0% to 87.6%)	
MRI position	Prone	90.1% (84.4% to 93.9%)	0.123	85.2% (79.6% to 89.5%)	0.120
	Supine	77.7% (54.7% to 91.0%)		74.3% (57.6% to 86.0%)	
Node selection	Largest node	96.2% (87.3% to 99.0%)	0.040	89.5% (78.2% to 95.3%)	0.194
	Other methods	86.0% (78.5% to 91.2%)		81.9% (75.5% to 86.9%)	
Reference test	Other	89.7% (82.1% to 94.3%)	0.575	84.6% (77.2% to 89.9%)	0.645
	SLNB or ALND	86.7% (75.1% to 93.4%)		82.3% (72.9% to 88.9%)	
Participant age	51 and over	82.5% (72.6% to 89.3%)	0.019	84.9% (77.8% to 90.0%)	0.551
	Less than 51	93.6% (87.9% to 96.8%)		81.8% (72.1% to 88.7%)	
Analysis type	Node by node	86.5% (75.4% to 93.1%)	0.494	83.9% (75.1% to 90.0%)	0.914
	Patient by patient	90.1% (82.1% to 94.8%)		83.4% (75.5% to 89.1%)	
MRI slice thickness	4.5 and above	84.3% (68.5% to 92.9%)	0.319	83.5% (73.4% to 90.3%)	0.711
	Less than 4.5 mm	90.4% (83.6% to 94.6%)		81.6% (74.8% to 86.8%)	
Repetition time	Less than 8500	88.1% (80.7% to 92.9%)	0.512	80.2% (74.3% to 85.0%)	0.080
	More than 8500	92.2% (75.9% to 97.8%)		90.8% (79.4% to 96.2%)	
	Less than 3000	78.8% (51.2% to 93.0%)	0.224	76.7% (59.6% to 88.0%)	0.394
	More than 3000	90.0% (83.9% to 94.0%)		82.9% (77.2% to 87.4%)	
Field of view	Less than 350	86.7% (77.2% to 92.6%)	0.335	77.6% (70.3% to 83.5%)	0.039
	350 and above	91.7% (82.4% to 96.3%)		87.3% (80.6% to 91.9%)	

Retrospective study design and FOV ≥ 350 mm had significantly higher specificities. While the use of 1000 s/mm^2^ as the largest *b*‐value and TR of more than 8500 ms was associated with higher specificities, the differences were marginally non‐significant. Selection of the largest node for evaluation and evaluation of participants younger than 51 years had significantly higher sensitivity.

### 3.7 AUC Meta‐Analysis

To confirm the primary results of our analysis, we meta‐analyzed the reported ROC AUCs as an index of the classification ability. The pooled AUC was 0.878 (CI = 0.843–0.914) which is in agreement with the main analysis. The forest plot for this analysis is available in Figure S1.

### 3.8 Benign and Metastatic Nodes ADC Values

**FIGURE 4:**
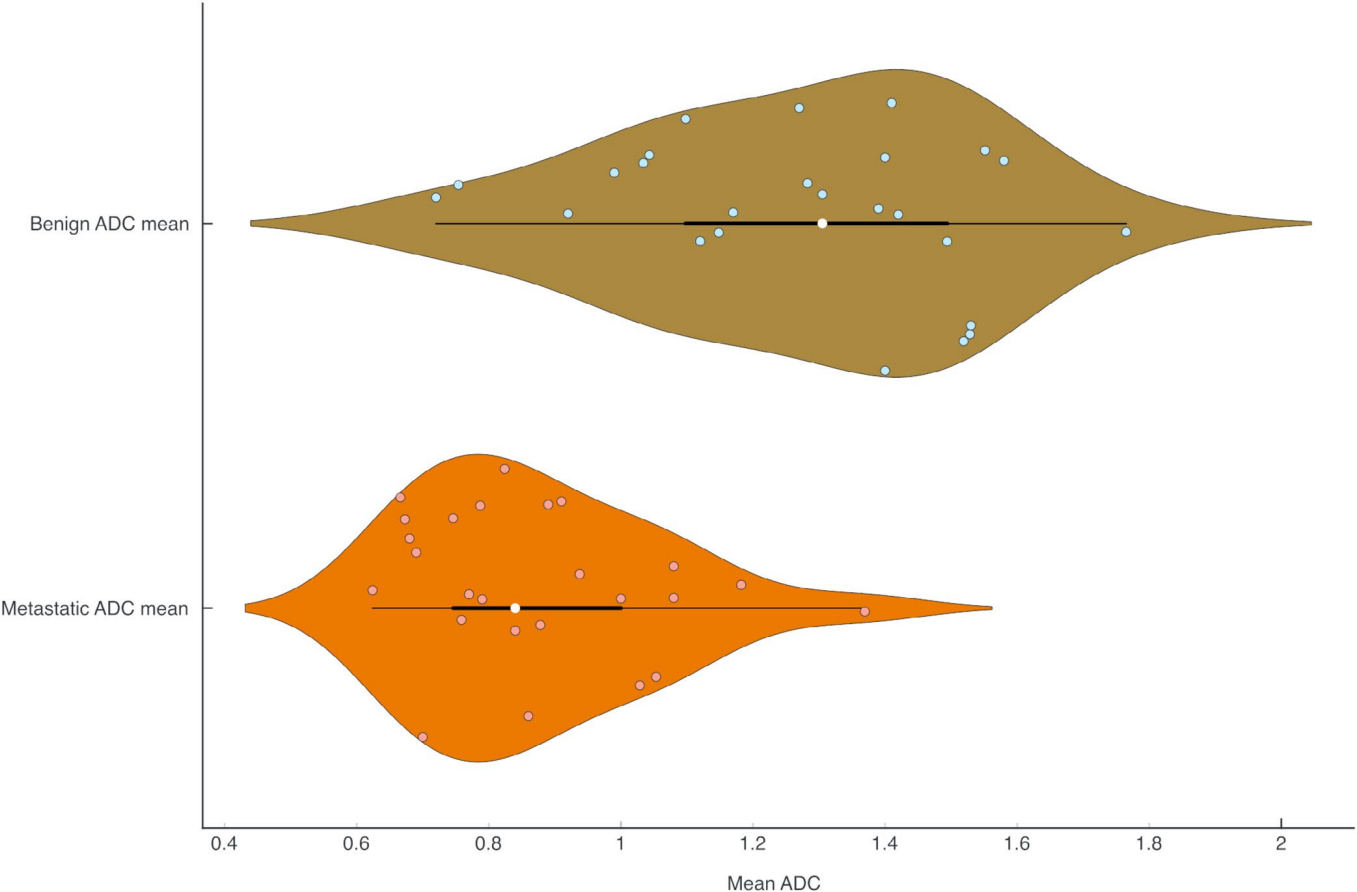
Violin and box plot of distribution of the apparent diffusion coefficient for benign and metastatic lymph nodes.

The mean ADC values for metastatic and non‐metastatic LNs were separately pooled and compared through a meta‐analysis of mean difference. A graphical depiction of the distribution of the ADC values is shown in Figure 4. The pooled ADC values of benign and metastatic LNs were 1.272 × 10^−3^ mm^2^/s (CI = 1.150–1.394) and 0.874 × 10^−3^ mm^2^/s (CI = 0.773–0.974), respectively. A significant mean difference of 0.395 × 10^−3^ mm^2^/s (CI = 0.311–0.479) is suggestive of lower ADC values in metastatic LNs which can be used for differentiating the two. The forest plot for this analysis along with the test statistics is available in Table S7 and Figure S2.

### 3.9 Summary of Findings

Certainty of evidence was assessed with GRADEpro. The evidence was judged to have a moderate level of overall certainty. The risk of bias scale was rated as severe due to the included studies' varying level of quality. Moderate heterogeneity was detected and the inconsistency scale was also marked as serious. The strong observed associations and lack of detectable publication bias were among the positive criteria in this assessment. This moderate certainty of evidence may warrant higher quality future studies with a specific focus on the limitations of previous studies. Summary of findings is presented in Table 4.

**TABLE 4:** Grade summary of evidence and evidence certainty.

Outcome	Number of studies (Number of patients)	Study design	Factors that may decrease certainty of evidence					Effect per 1000 patients tested			Test accuracy CoE
			Risk of bias	Indirectness	Inconsistency	Imprecision	Other considerations	Pre‐test probability of 15%	Pre‐test probability of 40%	Pre‐test probability of 60%	
True positives	26 (2828)	Cohort and case–control type studies	Serious	Not serious	Serious	Not serious	Strong association No publication bias	133 (124–139)	354 (330–371)	532 (494–557)	⊕⊕⊕◯ Moderate
False negatives								17 (11–26)	46 (29–70)	68 (43–106)	
True negatives	26 (2828)	Cohort and case–control type studies	Serious	Not serious	Serious	Not serious	Strong association No publication bias	711 (663–749)	502 (468–529)	334 (312–352)	⊕⊕⊕◯ Moderate
False positives								139 (101–187)	98 (71–132)	66 (48–88)	

### 3.10 Clinical Application

**FIGURE 5:**
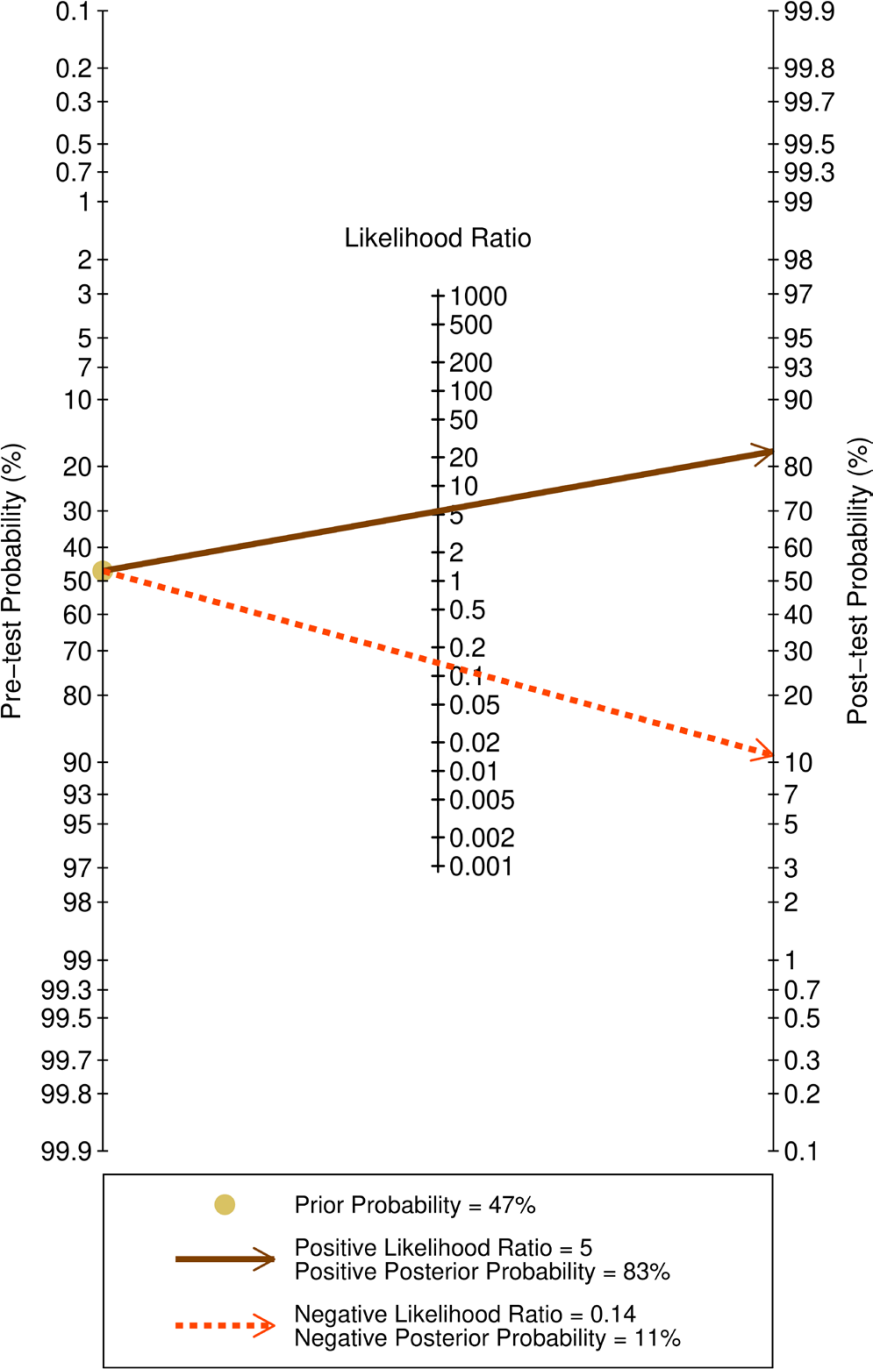
Fagen's nomogram for post‐test probability of the apparent diffusion coefficient in evaluating lymph node metastasis of breast cancer.

Fagen's nomogram for the estimation of posterior probabilities based on prior probabilities and test likelihood indices is shown in Figure 5.

## 4 Discussion

The use of DWI for breast cancer is on the rise. As many as 81% of radiologists employ some variant of DWI in their standard workups while technical and imaging parameters differ greatly [39]. This imaging technique can also be applied to axillary LNs with great diagnostic accuracy. And so, following the European Society of Breast Imaging (EUSOBI) consensus on the use of DWI in breast cancer, we set out to systematically gather relevant records pertaining to LN metastasis in breast cancer [40].

The pooled sensitivity and specificity were estimated to be 88.6% and 83.6%, respectively. The classification ability was considered extremely high with the SROC AUC of 0.92. Overall, the ADC value emerged as a highly accurate modality for diagnosis and prediction of breast cancer LN metastasis. Moderate heterogeneity was detected between the included studies (95% prediction area = 0.369, bivariate *I* ^2^ = 69.9%). After ruling out the threshold effect as the source of heterogeneity, meta‐regression and subgroup analysis were used to determine the origin of heterogeneity. An FOV of ≥ 350 mm and retrospective study design were identified as factors associated with higher specificity. Selection of the largest nodes in the DW image for analysis and participants aged < 51 years were factors associated with higher sensitivity. In our analysis, *b*‐values < 800 s/mm^2^ were highly sensitive for diagnosis of lymphatic metastasis. The 1000 s/mm^2^ *b*‐value achieved the greatest overall diagnostic odds ratio and was highly specific. EUSOBI has recommended the *b*‐value of 800 s/mm^2^ for DWI of primary breast tumors [40]. Our findings raise the interesting possibility that LN metastases and primary breast tumors may benefit from different DWI acquisition parameters to achieve optimal classification.

Given all the developments in non‐invasive diagnostic approaches, invasive procedures for the detection of metastatic LNs remain the most effective. Other modalities used for the evaluation of LN metastasis appear to have a wide range of diagnostic utilities. [^18^F]‐FDG PET/CT has demonstrated 58% and 83% sensitivity and specificity, respectively, while the sensitivity and specificity of [^18^F]‐FDG PET/MRI were reported to be 76% and 85% for the detection of breast cancer LN involvement [41]. In comparison, contrast enhanced ultrasound (CEUS) has demonstrated higher accuracy in the diagnosis of lymphatic metastasis (sensitivity 91%; specificity 86%) [42]. Although, as CEUS is based on the introduction of contrast agents in the body, hypersensitivity reactions may be a limiting factor. In a large network meta‐analysis, ultrasound elastography and MRI were reported to be the most sensitive modalities for identifying breast cancer LN metastasis. MRI achieved the highest relative sensitivity (1.13) and highest relative specificity (1.11) among all studied modalities [43].

Artificial intelligence (AI) augmented modalities may also be beneficial for enhancing detection accuracy. Computed tomography and MRI augmented with AI have shown 88% and 85% sensitivity, and 80% and 81% specificity for predicting breast cancer LN metastasis respectively [44]. Ultrasound imaging based on AI algorithms has also shown high accuracy in predicting breast cancer LN metastasis (sensitivity 88%; specificity 75%; AUC 0.89) [45]. Implementation of AI in DWI can be used for LN region‐of‐interest delineation or for constructing radiomics models which may in turn boost the diagnostic accuracy of this modality.

Regarding ADC values of breast lesions, a large‐scale meta‐analysis demonstrated that the ADC values of malignant breast lesions were significantly lower than those of benign lesions, irrespective of MRI magnetic strength, choice of *b*‐value, and the measurement method [10]. Additionally, it has been shown that tumors that are negative for human epidermal growth factor receptor‐2 and those that are positive for estrogen or progesterone receptors have lower ADC values of primary breast lesions [46]. The ADC value, however, was not a helpful indicator for distinguishing between the molecular subtypes of breast cancer in a multicenter study [47].

Contrasting evidence has been presented for the usefulness of the ADC value in other neoplastic lymphatic involvement. The ADC value has demonstrated promising diagnostic accuracy in the detection of lymphatic metastasis in cervical cancer [48, 49]. In squamous cell cancers of the head and neck, metastatic LNs can be differentiated using ADC values with very high sensitivity and specificity (90% and 88%) [50]. Contrastingly, the same cannot be said for colorectal lymphatic spread, where the use of ADC values of metastatic and non‐metastatic LNs resulted in poor diagnostic ability [51]. Studies have shown a negative correlation between ADC values and the Ki‐67 index and cell count of different tumors [52, 53]. Interestingly, the aforementioned reports also demonstrated diverse levels of correlation based on tumor type. This may help explain why lymphatic involvement in some tumor types can be distinguished based on the ADC values. Higher cellularity of lesions may lead to decreased intracellular and extracellular space and restricted water molecular diffusion, which is reflected as a lower ADC value.

The study limitations must be acknowledged. The presented results are a synthesis of previously published works; nevertheless, we strived to conduct a comprehensive search and the lack of detectable publication bias is a testament to this fact. Moderate heterogeneity was detected. Although some sources of heterogeneity were identified, still, the use of various imaging techniques restricts us from developing a comprehensive guideline for DWI of breast lymphatic metastasis. Furthermore, we acknowledge that potential variability in the specific formulas and software used to calculate the ADC maps across the included studies represents a limitation. This technical information was inconsistently reported, preventing its inclusion in our meta‐regression. Evidence certainty was deemed moderate owing to the suboptimal overall quality of the included studies; therefore, conducting further higher‐quality research on the topic is recommended. As we have demonstrated, the ADC value is a promising diagnostic marker of lymphatic metastasis and the development of a unified approach may lead to mass adoption. Future studies, adhering to the recommendations of this study, should explore the region of interest delineation, differences in minimum, maximum, or mean ADC values within ROIs, ratio of ipsilateral to contralateral axillary node ADC values, and node to tumor ADC ratio.

## 5 Conclusion

The ADC value obtained from diffusion imaging is a highly sensitive and specific marker of lymphatic involvement in breast cancer with very good diagnostic performance. Regarding imaging parameters, we recommend the use of at least two *b*‐values with the highest *b*‐value of 1000 s/mm^2^, TR of no less than 3000 ms and preferably more than 8500 ms, TE should be tuned to specific equipment but should be as low as possible, and the FOV should be 350 mm or above. Selection of the largest node for analysis seems to be associated with high sensitivity. Finally, DW imaging demonstrated the best performance in women under the age of 51.

## Author Contributions

**Amirmohammad Azizzadeh:** conceptualization (equal), data curation (equal), formal analysis (equal), methodology (equal), project administration (equal), visualization (equal), writing – original draft (equal), writing – review and editing (equal). **Fahimeh Zeinalkhani:** conceptualization (equal), project administration (equal), writing – original draft (equal), writing – review and editing (equal). **Peyman Kamali Hakim:** conceptualization (equal), writing – original draft (equal), writing – review and editing (equal). **Aida Mousavi:** data curation (equal), validation (equal), writing – original draft (equal), writing – review and editing (equal).

## Ethics Statement

This systematic review and meta‐analysis does not involve direct data collection from human participants. The research protocol was registered in PROSPERO (CRD42024556477). Ethical and methodological approval was obtained from the Internal Review Board (IR.TUMS.IKHC.REC.1403.244).

## Consent

The authors have nothing to report.

## Conflicts of Interest

The authors declare no conflicts of interest.

## Supporting information


**Data S1:** Supporting Information.

## Data Availability

The datasets generated or analyzed during the study are included in this published article and its supplement.
