# Supplementary material for: Diagnostic Test Accuracy of Apparent Diffusion Coefficient in Evaluation of Breast Cancer Lymph Node Metastasis: A Systematic Review and Meta‐Analysis
Source: Cancer Rep (Hoboken). 2025 Nov 8;8(11):e70395. doi: 10.1002/cnr2.70395 (PMC12595605; doi:10.1002/cnr2.70395)
Supplement: Supplementary file 1 — Data S1: Supporting Information. [file CNR2-8-e70395-s001.docx]

Supplementary materials

Supplementary appendix to:

Diagnostic test accuracy of apparent diffusion coefficient in evaluation of breast cancer lymph node metastasis: a systematic review and meta-analysis

Table of Contents

[Search strategy 2](#_Toc181825944)

[Supplementary table 1 PubMed search syntax 2](#_Toc181825945)

[Supplementary table 2 Web of science search syntax 3](#_Toc181825946)

[Supplementary table 3 EMBASE search syntax 4](#_Toc181825947)

[Included Studies’ characteristics 5](#_Toc181825948)

[Supplementary table 4 Study characteristics; imaging parameters 5](#_Toc181825949)

[Supplementary Table 5 Study characteristics; outcome measures 6](#_Toc181825950)

[Subgroup analysis of test accuracy based on b-value 7](#_Toc181825951)

[Supplementary Table 6 Subgroup analysis 7](#_Toc181825952)

[Meta-analysis of reported ROC AUC 8](#_Toc181825953)

[Supplementary Fig. 1 Forest plot of ROC AUC meta-analysis. 8](#_Toc181825954)

[Pooled ADC values for metastatic and benign nodes 9](#_Toc181825955)

[Supplementary Table 7 Pooled ADC and heterogeneity measures 9](#_Toc181825956)

[Mean difference meta-analysis of lymph node ADC values 10](#_Toc181825957)

[Supplementary Fig. 2 mean difference meta-analysis of ADC and subgroup analysis 11](#_Toc181825958)

# Search strategy

In this systematic review and meta-analysis, PubMed, EMBASE, Web of science core collection, PROQUEST through WOS, and preprints available in WOS were searched up to October 2023 using a broad search strategy. Detailed search strategy is available in Supplementary tables 1-3.

## **Supplementary table 1** PubMed search syntax

| # | *Search Details* | *Result* |
| --- | --- | --- |
| 1 | "Breast cancer"[All Fields] OR "Breast"[All Fields] OR "Breast Neoplasms"[MeSH Terms] OR "Breast cancer"[All Fields] OR "breast tumor"[All Fields] OR "breast carcinoma"[All Fields] OR "mammary cancer"[All Fields] OR "mammary carcinoma"[All Fields] OR "mammary neoplasms"[All Fields] OR "mastocarcinoma"[All Fields] OR "mastocarcinoma"[All Fields] | 635,223 |
| 2 | "Diffusion Magnetic Resonance Imaging"[MeSH Terms] OR "Diffusion MRI"[All Fields] OR "diffusion weighted mri"[All Fields] OR "DWI"[All Fields] OR "diffusion-weighted magnetic resonance imaging"[All Fields] OR "MRI-DWI"[All Fields] OR "diffusion-weighted imaging"[All Fields] OR "diffusion weighted mri"[All Fields] OR "apparent diffusion coefficient"[All Fields] OR "ADC value"[All Fields] OR "ADC"[All Fields] | 60,464 |
| 3 | "Lymph Nodes"[MeSH Terms] OR "Lymphatic System"[MeSH Terms] OR "Neoplasm Metastasis"[MeSH Terms] OR "Lymphatic Metastasis"[MeSH Terms] | 483,240 |
| 4 | "Lymph Nodes"[MeSH Terms] OR "Lymphatic System"[MeSH Terms] OR "Neoplasm Metastasis"[MeSH Terms] OR "Lymphatic Metastasis"[MeSH Terms] OR "axillary Lymph node"[All Fields] OR ("lymph*"[All Fields] AND "metastas*"[All Fields]) OR ("axilla*"[All Fields] AND "lymph*"[All Fields]) | 560,336 |
| 5 | ("Breast cancer"[All Fields] OR "Breast"[All Fields] OR "Breast Neoplasms"[MeSH Terms] OR "Breast cancer"[All Fields] OR "breast tumor"[All Fields] OR "breast carcinoma"[All Fields] OR "mammary cancer"[All Fields] OR "mammary carcinoma"[All Fields] OR "mammary neoplasms"[All Fields] OR "mastocarcinoma"[All Fields] OR "mastocarcinoma"[All Fields]) AND ("Diffusion Magnetic Resonance Imaging"[MeSH Terms] OR "Diffusion MRI"[All Fields] OR "diffusion weighted mri"[All Fields] OR "DWI"[All Fields] OR "diffusion-weighted magnetic resonance imaging"[All Fields] OR "MRI-DWI"[All Fields] OR "diffusion-weighted imaging"[All Fields] OR "diffusion weighted mri"[All Fields] OR "apparent diffusion coefficient"[All Fields] OR "ADC value"[All Fields] OR "ADC"[All Fields]) AND ("Lymph Nodes"[MeSH Terms] OR "Lymphatic System"[MeSH Terms] OR "Neoplasm Metastasis"[MeSH Terms] OR "Lymphatic Metastasis"[MeSH Terms] OR "axillary Lymph node"[All Fields] OR ("lymph*"[All Fields] AND "metastas*"[All Fields]) OR ("axilla*"[All Fields] AND "lymph*"[All Fields])) | 209 |

## **Supplementary table 2** Web of science search syntax

| # | Search Query | Results |
| --- | --- | --- |
| 1 | TS=((“Breast cancer” OR “Breast” OR “Breast Neoplasms” OR “breast tumor” OR “breast carcinoma” OR “mammary cancer” OR “mammary carcinoma” OR “mammary neoplasms” OR “mastocarcinoma”) AND (“Diffusion Magnetic Resonance Imaging” OR “Diffusion MRI” OR “diffusion weighted mri” OR “DWI” OR “diffusion-weighted magnetic resonance imaging” OR “MRI-DWI” OR “diffusion-weighted imaging” OR “diffusion weighted MRI” OR “apparent diffusion” OR “apparent diffusion coefficient” OR “ADC value” OR “ADC”) AND (“Lymph Nodes” OR “Lymphatic System” OR “Neoplasm Metastasis” OR “Lymphatic Metastasis”)) | 123 |
| 2 | ALL=("Breast cancer") OR ALL=(“Breast Neoplasms”) OR ALL=("Breast") OR ALL=(Breast*) OR ALL=("breast tumor") OR ALL=("breast carcinoma") OR ALL=("mammary cancer") OR ALL=("mammary carcinoma") OR ALL=("mammary neoplasms") OR ALL=("mastocarcinoma") | 873,508 |
| 3 | ALL=("Lymph Node") OR (ALL=(lymph*) AND ALL=(Metastasis)) OR ALL=("Lymphatic Metastasis") OR ALL=("axillary Lymph node") OR ALL=("axilla* lymph*") | 207,499 |
| 4 | ALL=("Diffusion Magnetic Resonance Imaging") OR (ALL=("Diffusion") AND ALL=("MRI")) OR ALL=("diffusion weighted mri") OR ALL=("DWI") OR ALL=("diffusion-weighted magnetic resonance imaging") OR ALL=("MRI-DWI") OR ALL=("DWI MRI") OR ALL=("diffusion-weighted imaging") OR ALL=("apparent diffusion") OR ALL=("apparent diffusion coefficient") OR ALL=("ADC value") OR (ALL=(ADC) AND ALL=(MRI)) | 63,109 |
| 5 | #2 AND #3 AND #4 | 258 |
| 6 | ALL=((“Breast cancer” OR “Breast” OR “Breast Neoplasms” OR “breast tumor” OR “breast carcinoma” OR “mammary cancer” OR “mammary carcinoma” OR “mammary neoplasms” OR “mastocarcinoma”) AND (“Diffusion Magnetic Resonance Imaging” OR “Diffusion MRI” OR “diffusion weighted mri” OR “DWI” OR “diffusion-weighted magnetic resonance imaging” OR “MRI-DWI” OR “diffusion-weighted imaging” OR “diffusion weighted MRI” OR “apparent diffusion” OR “apparent diffusion coefficient” OR “ADC value” OR “ADC”) AND (“Lymph Nodes” OR “Lymphatic System” OR “Neoplasm Metastasis” OR “Lymphatic Metastasis”)) | 127 |
| 7 | (#6) NOT #5 | 23 |
| 8 | #5 OR #7 | 281 |

## **Supplementary table 3** EMBASE search syntax

| # | Search Query | Results |
| --- | --- | --- |
| 1 | 'breast cancer'/exp OR 'breast carcinoma'/exp OR 'mastocarcinoma' OR 'breast tumor'/exp OR 'breast neoplasm' OR 'mammary cancer' OR 'mammary carcinoma' | 682,426 |
| 2 | 'diffusion weighted imaging'/exp OR 'diffusion mri' OR 'diffusion weighted mri' OR 'diffusion-weighted magnetic resonance imaging' OR 'mri-dwi' OR 'diffusion-weighted imaging' OR 'apparent diffusion coefficient'/exp OR 'apparent diffusion coefficient' OR 'adc value' OR ('adc' AND 'mri') | 73035 |
| 3 | 'lymph node'/exp OR 'axillary lymph node'/exp OR 'lymphatic system metastasis'/exp OR 'lymph node metastasis'/exp OR 'axillary lymph node metastasis'/exp OR 'neoplasm metastasis' OR 'lymphatic metastasis' OR ('axilla*' AND 'lymph*') | 385,287 |
| 4 | ('breast cancer'/exp OR 'breast carcinoma'/exp OR 'mastocarcinoma' OR 'breast tumor'/exp OR 'breast neoplasm' OR 'mammary cancer' OR 'mammary carcinoma') AND ('diffusion weighted imaging'/exp OR 'diffusion MRI' OR 'diffusion weighted MRI' OR 'diffusion-weighted magnetic resonance imaging' OR 'MRI-DWI' OR 'diffusion-weighted imaging' OR 'apparent diffusion coefficient'/exp OR 'apparent diffusion coefficient' OR 'ADC value' OR ('ADC' AND 'MRI')) AND ('lymph node'/exp OR 'axillary lymph node'/exp OR 'lymphatic system metastasis'/exp OR 'lymph node metastasis'/exp OR 'axillary lymph node metastasis'/exp OR 'neoplasm metastasis' OR 'lymphatic metastasis' OR ('axilla*' AND 'lymph*')) | 411 |

# Included Studies’ characteristics

## **Supplementary table 4** Study characteristics; imaging parameters

| ID | Study | Field strength | b-values | Slice thickness | TR | Field of view | DWI before contrast | MRI position | node selection | Analysis |
| --- | --- | --- | --- | --- | --- | --- | --- | --- | --- | --- |
| 1 | Başara | 1.5 T | 0, 600 | 5 | 7900 | 380 | Yes | Prone | All suspicious nodes | Node by node |
| 2 | Chung | 1.5 and 3.0 T | 0, 1000 | 3 | 9098 | 330 | Yes | Prone | Largest node | Patient by patient |
| 3 | De Cataldo | 1.5 T | 0, 800 | 3 | 4000 | 340 | Yes | Supine | Most clinically suspicious | Patient by patient |
| 4 | Duran | 1.5 T | 0, 1000 | 2 | 1000 | 320 | Yes | Prone | Most clinically suspicious | Patient by patient |
| 5 | Elmesidy | 1.5 T | 0, 200, 400, 1000 | 6 | 5000 | 300 | Yes | Supine | Most clinically suspicious | Patient by patient |
| 6 | Fardanesh | 3.0 T | 0, 800 | 3.9 | 6000 | 330 | Yes | Prone | Most clinically suspicious | Patient by patient |
| 7 | Fornasa | 1.5 T | 0, 800 | 4 | 4000 | 340 | Yes | Prone | Most clinically suspicious | Patient by patient |
| 8 | Guvenc | 1.5 and 3.0 T | 0, 1000 | NA | NA | NA | Yes | Prone | All suspicious nodes | Node by node |
| 9 | Hasanzadeh | 1.5 T | 50, 400, 800 | 5 | 7700 | 380 | Yes | Prone | All suspicious nodes | Node by node |
| 10 | He (a) | 1.5 T | 0, 500 | 4 | 5000 | 340 | Yes | Prone | All suspicious nodes | Node by node |
| 11 | He (b) | 1.5 T | 0, 800 | 4 | 5500 | 340 | Yes | Prone | All suspicious nodes | Node by node |
| 12 | Ismail | 1.5 T | 0, 750 | 5 | 7000 | 360 | Yes | Prone | Most clinically suspicious | Patient by patient |
| 13 | Kamitani | 1.5 T | 0, 1000 | 5 | 4600 | 360 | Yes | Prone | Largest node | Node by node |
| 14 | Kim | 3.0 T | 0, 750 | 4 | 5600 | 360 | Yes | Prone | Most clinically suspicious | Patient by patient |
| 15 | Kurt (a) | 1.5 T | 0, 800 | 3 | 5000 | 360 | Yes | Prone | Largest node | Patient by patient |
| 16 | Kurt (b) | 1.5 T | 0, 500 | 3 | 5000 | 360 | Yes | Prone | Largest node | Patient by patient |
| 17 | Latif | 1.5 T | 0, 500, 1000 | 5 | 1050 | 360 | Yes | Supine | Largest node | Patient by patient |
| 18 | Luo | 1.5 T | 0, 800 | 6 | 5800 | 320 | Yes | Prone | All suspicious nodes | Node by node |
| 19 | Razek | 1.5 T | 0, 500, 1000 | 4 | 10000 | 330 | Yes | Prone | All suspicious nodes | Node by node |
| 20 | Scaranelo | 1.5 T | 50, 300, 700, 1000 | 4 | 5340 | 320 | Yes | Prone | Most clinically suspicious | Patient by patient |
| 21 | Schipper (a) | 3.0 T | 0, 500, 800 | 3 | 2108 | 220 | Yes | Supine | All suspicious nodes | Node by node |
| 22 | Schipper (b) | 3.0 T | 0, 500, 800 | 3 | 2108 | 220 | Yes | Supine | All suspicious nodes | Patient by patient |
| 23 | Yamaguchi | 1.5 and 3.0 T | 0, 800 | 2.5 | 5669 | 360 | No | Prone | All suspicious nodes | Patient by patient |
| 24 | Yilmaz | 1.5 T | 0, 1000 | 4.5 | 8500 | 330 | Yes | Prone | Most clinically suspicious | Patient by patient |
| 25 | Zahran | 1.5 T | 0, 750 | 3 | 10036 | 340 | Yes | Prone | All suspicious nodes | Node by node |
| 26 | Zaiton | 1.5 T | 0, 1000 | 4 | 5700 | 360 | No | Prone | All suspicious nodes | Node by node |

## **Supplementary Table 5** Study characteristics; outcome measures

| ID | Study | TP | FP | FN | TN | Sensitivity | Specificity |
| --- | --- | --- | --- | --- | --- | --- | --- |
| 1 | Başara | 34 | 41 | 11 | 101 | 75.56 | 71.13 |
| 2 | Chung | 68 | 7 | 0 | 35 | 100.0 | 83.33 |
| 3 | De Cataldo | 41 | 8 | 9 | 49 | 82.61 | 85.96 |
| 4 | Duran | 54 | 5 | 10 | 33 | 84.38 | 86.84 |
| 5 | Elmesidy | 36 | 11 | 11 | 19 | 76.60 | 63.33 |
| 6 | Fardanesh | 76 | 23 | 31 | 87 | 71.03 | 79.09 |
| 7 | Fornasa | 18 | 2 | 1 | 22 | 94.74 | 91.67 |
| 8 | Guvenc | 35 | 3 | 7 | 91 | 83.33 | 97.85 |
| 9 | Hasanzadeh | 20 | 3 | 3 | 24 | 86.96 | 88.89 |
| 10 | He (a) | 130 | 53 | 4 | 64 | 97.01 | 54.70 |
| 11 | He (b) | 127 | 40 | 7 | 77 | 94.78 | 65.81 |
| 12 | Ismail | 32 | 2 | 0 | 10 | 100.0 | 87.00 |
| 13 | Kamitani | 14 | 11 | 12 | 73 | 53.85 | 86.90 |
| 14 | Kim | 69 | 26 | 22 | 136 | 75.82 | 83.95 |
| 15 | Kurt (a) | 42 | 2 | 1 | 21 | 97.67 | 91.30 |
| 16 | Kurt (b) | 42 | 2 | 1 | 21 | 97.67 | 91.30 |
| 17 | Latif | 18 | 1 | 0 | 11 | 100.0 | 91.67 |
| 18 | Luo | 37 | 6 | 8 | 28 | 82.22 | 82.35 |
| 19 | Razek | 41 | 0 | 3 | 21 | 93.18 | 100.0 |
| 20 | Scaranelo | 24 | 15 | 4 | 22 | 85.71 | 59.46 |
| 21 | Schipper (a) | 9 | 41 | 13 | 72 | 40.91 | 63.72 |
| 22 | Schipper (b) | 8 | 14 | 4 | 24 | 66.67 | 63.16 |
| 23 | Yamaguchi | 14 | 4 | 2 | 16 | 85.00 | 81.00 |
| 24 | Yilmaz | 12 | 2 | 8 | 21 | 60.00 | 91.30 |
| 25 | Zahran | 26 | 2 | 3 | 13 | 89.66 | 86.67 |
| 26 | Zaiton | 119 | 5 | 7 | 77 | 94.44 | 93.90 |

# Subgroup analysis of test accuracy based on b-value

Subgroup analysis of diagnostic test accuracy of the ADC value for detection of nodal metastasis is presented in Supplementary Table 6. Use of the b-value of 1000 s/mm^2^ resulted in the highest diagnostic odds ratio. B-values of less than 800 demonstrated the highest sensitivity.

## **Supplementary Table 6** Subgroup analysis of diagnostic test accuracy of ADC for evaluation of lymph node metastasis in breast cancer

| ADC value | Less than 800 | 800 | 1000 |
| --- | --- | --- | --- |
| Sensitivity | 93% (81.5-97.6%) | 85.2% (73.4-92.3%) | 85.2% (73.4-92.3%) |
| Specificity | 77.9% (64.9-87.1%) | 79.5% (71.4-85.8%) | 89.1% (79.8-94.4%) |
| DOR | 46.83 (13.37-163.98) | 22.24 (8.22-60.17) | 62.79 (19.24-204.88) |
| LR+ | 4.217 (2.538-7.006) | 4.15 (2.76-6.23) | 8.11 (4.16-15.80) |
| LR- | 0.09 (0.032-0.252) | 0.19 (0.10-0.36) | 0.13 (0.06-0.28) |

# Meta-analysis of reported ROC AUC

Reported areas under the receiver operating characteristics curve (ROC AUC) were pooled using the random effects model in MedCalc version 23.0. standard errors of AUC were calculated using the Hanley method. Pooled AUC was 0.878 (CI = 0.843 to 0.914) with I^2^ equaling 81.98%. The results were in agreement with the primary analysis results and confirmed its validity. The forest plot is presented in Supplementary figure 1.


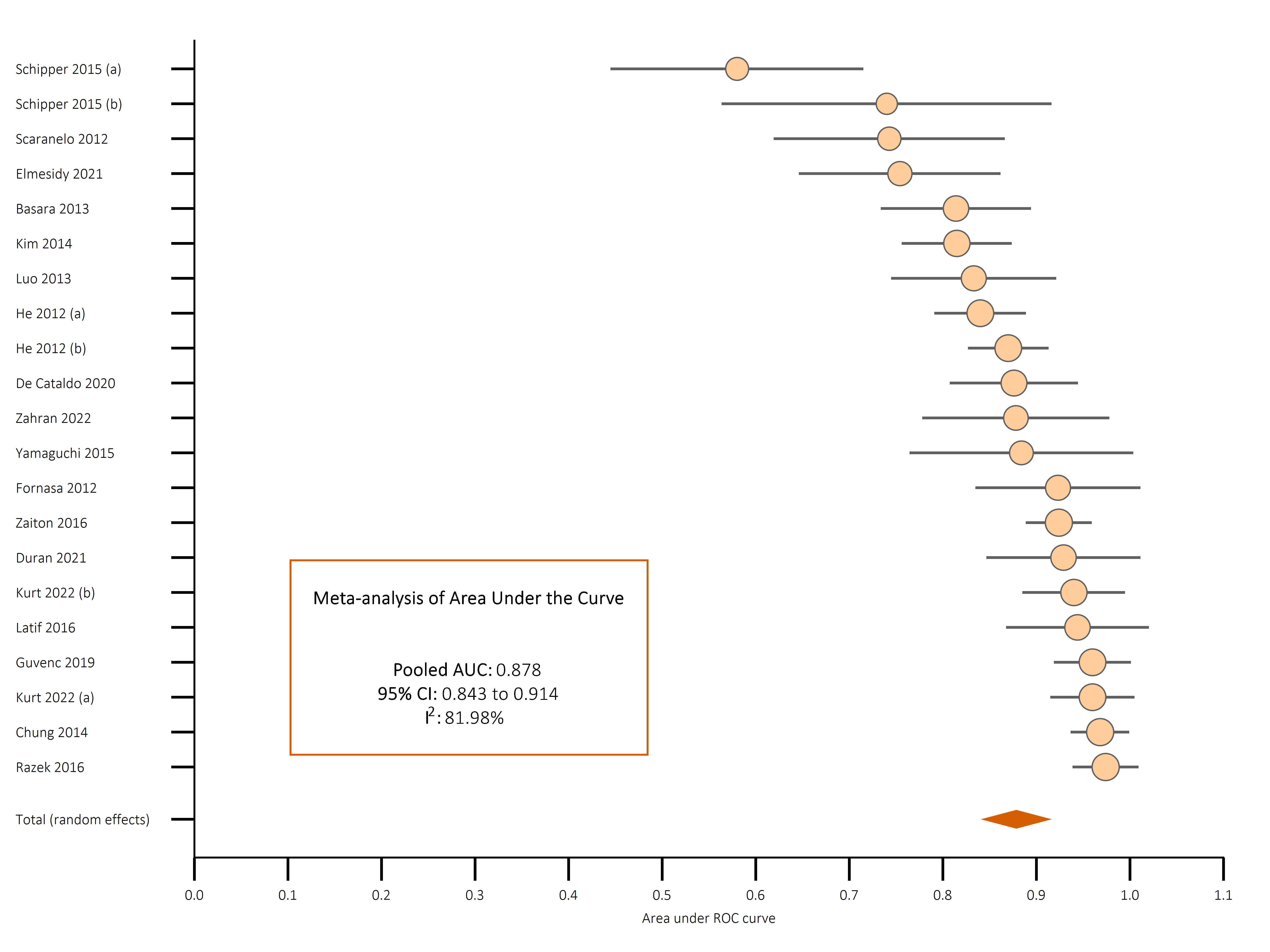


## **Supplementary Fig. 1** Forest plot of ROC AUC meta-analysis.

# Pooled ADC values for metastatic and benign nodes

Mean ADC values for metastatic and benign lymph nodes were pooled using the random effects model in Onlinemeta. Mean ADC were 1.272 x 10^-3^ mm^2^/s (CI = 1.150 to 1.394) for benign lymph nodes and 0.874 x 10^-3^ mm^2^/s (CI = 0.773 to 0.974) for metastatic lymph nodes. Substantial Heterogeneity was present in both models (I^2^ = 99%).

## **Supplementary Table 7** Pooled apparent diffusion coefficient and heterogeneity measures using random effects meta-analysis.

|  | Pooled Effect size | | | Heterogeneity | | | | |
| --- | --- | --- | --- | --- | --- | --- | --- | --- |
|  | N | Mean | 95% CI | Q-value | df (Q) | P-value | I^2^ | Tau^2^ |
| Benign nodes | 25 | 1.272 | 1.150 to 1.394 | 1684.79 | 24 | <0.001 | 99% | 0.094 |
| Metastatic nodes | 25 | 0.874 | 0.773 to 0.974 | 2327.90 | 24 | <0.001 | 99% | 0.064 |

# Mean difference meta-analysis of lymph node ADC values

A mean difference meta-analysis using the random-effects meta-analytical model was conducted in Onlinemeta. The overall mean difference was -0.395 with a 95% CI of -0.479 to -0.311, indicating a significant difference favoring lower ADC values in metastatic lymph nodes. Heterogeneity among the studies was substantial (Tau^2^ = 0.041; Chi^2^ = 378.67, df = 24, P < 0.01; I^2^ = 94%), suggesting variability in the effect sizes across studies. Despite this heterogeneity, the overall effect was statistically significant (Z = 9.22, P < 0.01). Subgroup analyses based on different b-values also showed consistent results, with similar and significant mean differences in ADC values between metastatic and benign lymph nodes across all subgroups (Chi^2^ = 0.54 , df = 3, P = 0.91). This reinforces the robustness of ADC as a diagnostic tool for differentiating between these conditions irrespective of the used b-value.


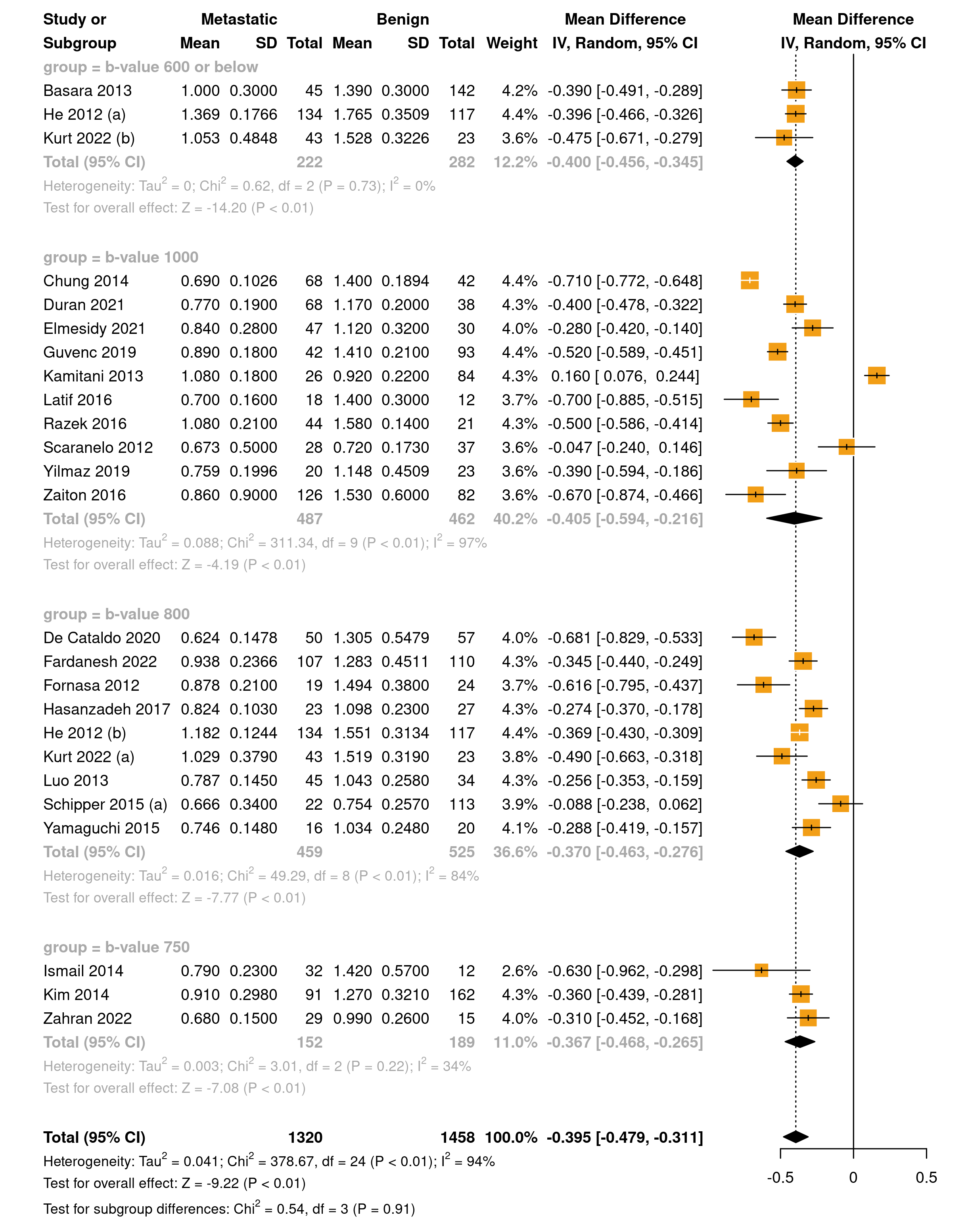


## **Supplementary Fig. 2** Forest plot of mean difference meta-analysis of reported ADC values for benign and metastatic nodes; subgroup analysis based on the largest utilized b-value is shown.
